# “*Cool*” and “*Hot*” Executive Functions in Patients With a Predominance of Negative Schizophrenic Symptoms

**DOI:** 10.3389/fpsyg.2020.571271

**Published:** 2020-11-05

**Authors:** Pamela Ruiz-Castañeda, Encarnación Santiago-Molina, Haney Aguirre-Loaiza, María Teresa Daza González

**Affiliations:** ^1^Neuropsychological Evaluation and Rehabilitation Center, University of Almería, Almeria, Spain; ^2^Department of Psychology, University of Almería, Almeria, Spain; ^3^Hospital Day of the Mental Health Clinical Management Unit, Almeria, Spain; ^4^Department of Psychology, Catholic University of Pereira, Pereira, Colombia

**Keywords:** hot executive functions, psychosis, frontosubcortical syndromes, dysexecutive syndrome, negative symptoms, cool executive function

## Abstract

**Background:**

Patients with psychosis often present significant neurocognitive deficits, with executive function deficits (EEFF) being one of the most relevant cognitive impairments with the greatest impact on the functioning of their daily lives. However, although various findings of executive involvement were reported, it is not entirely clear whether there is a differential pattern of involvement according to the clinical symptoms or the deficits occur in all or only in some subcomponents of EEFF.

**Objective:**

The present study had a double objective: to study the specific deficits in the cool and hot EEFF in a group of psychotic patients with a predominance of negative symptoms; and determine the possible associations between the performance of the patients in the cool an hot EEFF tasks with the negative symptoms, and with the behavioral alterations associated with the dysexecutive syndrome.

**Method:**

66 participants, 33 psychotic patients with a predominance of negative symptoms and 33 healthy control subjects matched in gender, age and educational level participated. Both groups were administered 4 cool EEFF tasks (coding/maintenance and updating of information in working memory, ability to change the mental set and planning), and 3 hot EEFF tasks (decision making in situations of uncertainty, recognition of emotions through facial expressions and theory of mind). In the group of patients, the Negative symptoms were evaluated through the Scale for the Evaluation of Negative Symptoms (SANS), and the behavioral alterations associated with dysexecutive syndrome through the subscale of “Executive Dysfunction” of the Frontal Systems Behavior Scale.

**Results:**

Patients performed worse on three *cool* EEFF tasks and on two of the *hot* EEFF tasks. Additionally, we found a correlation between the SANS score and the “executive dysfunction” subscale, with the cold EEFF task that measures planning.

**Conclusion:**

Our findings showed that in psychotic patients with a predominance of negative symptoms, both, the cognitive (cool) and emotional (hot) components of executive functions are affected. The results reinforce the need for a cognitive rehabilitation treatment of the executive components of the working memory and of those more socio-emotional aspects.

## Introduction

Negative symptoms (NS) have been considered as a central characteristic of psychosis, constituting a serious cause of disability and having a clear impact on the patient’s daily life functioning (Fonseca et al., 2015). Patients with predominantly NSs (affective flattening, alogia, abulia or apathy, anhedonia and asociality) have been associated with considerable cognitive impairment, specifically, some previous studies have found deficits in Executive Functions (EEFF) (v.g. Martino et al., 2007; Jang et al., 2017; Avcu et al., 2019). However, it is important to note that in all previous studies the EEFF are not defined in the same way or measured with the same instruments.

Traditionally, the Executive Functions (EEFF) have been considered as a term that brings together a series of higher order processes that allow us to carry out actions aimed at a goal and to provide adaptive responses to novel or complex situations. The EEFF are difficult to define as an unitary entity, so the distinction between a cognitive or *cool* component of EEFF and an emotional or *hot* component has been suggested, considering both components as two sets of interrelated but distinguishable processes both functionally and anatomically (see Table 1).

**TABLE 1:** *Cool* and *hot* components of executive functions (adapted from Brock et al., 2009).

**Executive functions**		
Component:	***cool (cognitive)***	***hot (emotional)***
	Work memory Inhibition Cognitive flexibility Planning	Decision making Theory of mind Events with emotional consequence
Neuroanatomical area	Dorsolateral PFC and the lateral parietal cortex	Ventromedial PFC, and anterior cingulate cortex
Deficit	It is associated with the loss of the ability to learn new information, difficulties in solving problems and in finding novel solutions.	It is associated with impulsivity problems, inability to participate in perspective taking, and the inappropriate social behavior.

*PFC, prefrontal cortex.*

According to this distinction, cognitive or *cool* EEFF refer to those processes involved in solving abstract and decontextualized problems, without any affective component or social interaction. In contrast, emotional or *hot* EEFF refers to those processes involved in contexts that generate emotion, motivation, and tension between immediate gratification or greater reward in the longer term, also being important for our social interactions.

Respect to *cool* EEFF, there is some agreement among researchers that there would be at least three central components: (1) the coding/maintenance and updating processes of the information in working memory; (2) inhibitory control; and (3) the cognitive flexibility or ability to change the mental set (Miyake et al., 2000). Based on these central cognitive EEFF, other more complex ones would be developed such as planning, abstract reasoning or problem solving. In contrast, the organization of the *hot* EEFF is less known, but there is some agreement that these functions would be involved, at least, in decision-making in situations of uncertainty, the recognition of facial expressions and their emotional content, as well as in the ability to infer the perspective of others also known as theory of mind (ToM). Therefore, a well-orchestrated functioning of all these *cool* and *hot* EEFF will be crucial for the activities of daily life and for our social relationships, since these would be the functions that direct our behavior (self-regulation) and our emotional, social and cognitive activity.

However, most of the previous neuropsychological studies that have studied the deficits in EEFF of psychotic patients with NS have focused almost exclusively on *cool* EEFF (v.g. Meiran et al., 2000; Jogems-Kosterman et al., 2001; Menon et al., 2001; Henik et al., 2002; Donohoe and Robertson, 2003; Rocca et al., 2009; Liemburg et al., 2015).

Previous studies that have been interested in *hot* EEFF, such as decision-making in situations of uncertainty (Bechara et al., 1994, 1999, 2002; Young et al., 2010) or the ToM (Catalan et al., 2018; Thibaudeau et al., 2019), are much scarcer, and in some of them the results obtained are contradictory (Wilder et al., 1998; Brüne, 2001, 2003; Ritter et al., 2004; Peyroux et al., 2019).

Therefore, to date, no conclusive results have been obtained regarding the specific deficits that patients with negative schizophrenic symptoms present in the EEFF, whether there is a greater deterioration in executive functions of a more cognitive type or whether, on the contrary, there could be an impairment in executive functions of more socio-emotional type.

From a neuroanatomical point of view, EEFF have been directly related to an adequate functioning of the prefrontal cortex (PFC). *Cool* EEFF have been related to dorsolateral PFC and the *hot* EEFF have been associated with the activity of the orbitofrontal and ventromedial regions of the PFC, two regions of the brain largely overlapping and strongly connected to the limbic areas associated with emotional and social processing (Happaney et al., 2004).

This neuroanatomical differentiation is important since in the scientific literature, we can find several studies that suggest that NS could be a clinical manifestation of dysfunction of the prefrontal cortex (e.g., Addington et al., 1991; Weinberger et al., 1992; Berman et al., 1997; Callicott, 2000; Takizawa et al., 2008; Shimodera et al., 2012). In fact, there is a certain similarity between NS (affective flattening, allogy, abulia or apathy, anhedonia and asociality) and the dysfunctions that have been described in patients with damage to the prefrontal cortex (PFC). Thus, for example, Castaño et al. (2012) found that a significant percentage of patients with lesions in the prefrontal cortex (49%), also presented psychopathological alterations such as emotional lability, affective flattening, apathy, or decreased initiative.

However, it is not clear whether NS could be primarily related to dysfunction in the dorsolateral region of the PFC or whether it could also reflect dysfunction of the ventromedial and orbitofrontal regions. From our study and also given the importance of the different regions of the PFC in the functioning of the EEFF, the analysis of the specific deficits in the *hot* and cold EEFF in patients with negative schizophrenic symptoms, offers the possibility of exploring the relationship between the NS and the possible prefrontal dysfunction, being able to further investigate whether the different PFC regions associated with EEFF (dorsolateral and ventromedial/orbitofrontal) could be equally affected in these patients.

In summary, and based on the knowledge provided by studies in the scientific literature on NS, we consider that there are a series of reasons that justify the importance of studying the possible relationships of NSs with the dysfunctionality of the EEFF and the behavioral components of the Dysexecutive syndrome, namely:

(1) To deepen the study of the EEFF of patients with a predominance of negative schizophrenic symptoms, addressing both, the *cold* and *hot* components traditionally less attended. Likewise, it is interesting to explore the relationship between the severity of the NS and the different components of the EEFF and if the clinical variables (clinical setting to which the patient belonged -treatment in hospital or outpatient regimen-, duration of illness and pharmacological treatment) are related to patient performance.
(2) Regarding the evaluative instruments of the EEFF used in most neuropsychological studies, these have been not very specific and very diverse, making it difficult to compare results between them. So we propose to use execution tasks based on experimental paradigms of cognitive neuroscience as evaluative instruments. The advantage and novelty that this report, is that they are evaluation instruments that allow us to obtain finer and more precise measurements for the study of EEFF.
(3) Given the importance of the different regions of the PFC in the functioning of the EEFF, the study of the specific deficits in the *hot* and *cold* EEFF in patients with a predominance of negative schizophrenic symptoms offers the possibility of investigating the relationship between NS and prefrontal dysfunction, being able to investigate whether the different regions of the PFC (dorsolateral and ventromedial/orbitofrontal) could be equally affected in these patients, this, when observing the behavioral deficits that the scientific literature relates to the affectation of the different regions of the PFC.

In this sense, the present study had a double objective. First, to study the specific deficits in the *cool* and *hot* EEFF in a group of psychotic patients with a predominance of NSs. For this, different execution tasks based on experimental paradigms of cognitive neuroscience were used, which have been shown to be sensitive to detect dysfunctions in the different regions of the PFC. Specifically, 4 *cool* EEFF tasks associated with dorsolateral PFC were used (coding/maintenance and updating of information in working memory, ability to change the mental set and planning), and 3 EEFF *hot* tasks associated with the orbitofrontal and ventromedial regions of the PFC. Patient performance was compared to that of a control group of healthy subjects matched for age, gender, and educational level. It was also explored if in the group of patients, the main clinical variables (duration of the disease, clinical setting to which the patient belonged -treatment in hospital or outpatient regimen-, and pharmacological treatment), influenced the execution of the tasks of EEFF. Secondly, the degree of correlation between the severity of the NS (measured through the “Scale for the Evaluation of NSs -SANS-”) (Andreasen, 1989) and the performance in the tasks of *cool* (dorsolateral PFC) and *hot* (Ventromedial and orbitofrontal PFC) EEFF was determined. Additionally, it was also explored in the group of patients if the execution in the *cool* EEFF tasks correlated with the behavioral alterations associated with the dorsolateral prefrontal syndrome or dysexecutive syndrome, a syndrome that, in patients with brain damage, has been associated with damage in dorsolateral PFC (Keefe et al., 1992; Dolan et al., 1993; Wilder et al., 1998). To obtain a measure of these dysexecutive behaviors in psychotic patients, the executive dysfunction subscale of the Frontal Systems Behavior Scale -FrSBe- was used (Grace and Malloy, 2001; Pedrero et al., 2009).

Considering the previous literature, regarding our first objective, we expect that psychotic patients with a predominance of NSs show a significantly lower performance than the control group in the EEFF tasks, especially in the *cool* EEFF tasks. On the other hand, regarding the clinical variables, we hope that the duration of illness and the type of neuroleptic treatment may be related to the performance of the EEFF tasks.

Regarding the second objective, if the NS are a clinical manifestation of a dysfunction mainly in the dorsolateral region of the PFC, we expect that patients with higher scores on the Scale for the Evaluation of Negative Symptoms (SANS) present a lower performance in *cool* EEFF tasks, whereas we don’t expect to find any correlation with execution in *hot* EEFF tasks. Likewise, it would be expected that the patients who present more behaviors associated with Dorsolateral Prefrontal Syndrome, are those that also show a worse execution in the specific tasks of *cool* EEFF.

## Materials and Methods

### Participants

**FIGURE 1:**
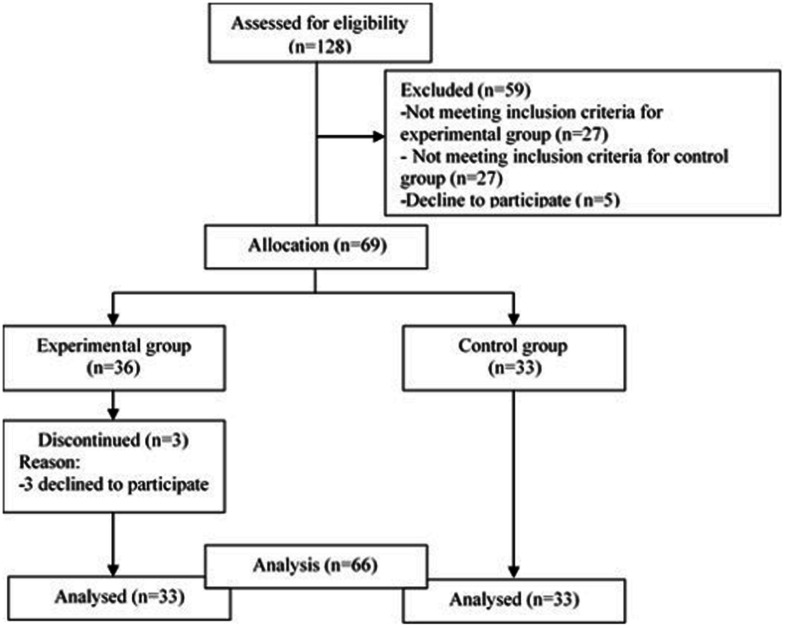
Flow of participants throughout the study.

The initial sample consisted of 129 participants (age range min = 20 – max = 61, *M*_*age*_ = 40.9, *SD* = 11.17). The process of choosing and selecting is shown in Figure 1. With respect to the experimental group, psychotic patients were included in the study with a definitive diagnosis of psychosis (paranoid schizophrenia or schizoaffective disorder), and with a confirmed diagnosis with 2 years of evolution, as well as patients with a predominance of NSs, these being the patients who presented a higher percentage in the Scale for the Assessment of Negative Symptoms (SANS) than the Scale for the Assessment of Positive Symptoms (SAPS). Likewise, patients with a stable psychopathological state that would allow the tests to be carried out. The criterion of psychopathological stability that allowed us to perform the neuropsychological evaluation was established by the psychiatrist of reference, based on his knowledge of the patient’s clinic and always ensuring a compensated state during the last months prior to the evaluation, as well as a motivation active for participation in the study. The patients were selected from the different medical devices of the Mental Health area of the reference Hospital Complex of the city. Respect to the control group, healthy subjects matched to the patient group in age, gender, and years of schooling were recruited; no history of mental or neurological illness, substance use disorders, and they were not taking psychotropic medications. Before the study was carried out, the approval of the Research Ethics Committee of the hospital to which the patients belonged was obtained, respecting the ethical principles of the 2013 Helsinki declaration and other international codes. All participants gave their written informed consent to participate.

In the group of patients, regarding the sociodemographic variables, three levels were established according to the years of education: basic (6 years), medium (between 7 and 12 years), high (over 12 years). Respect the clinical variables, for the duration of illness, two levels were established according to the sample mean: a group with a shorter duration off illness (less than 11 years) and another group with a longer duration of illness (more than 11 years). Regarding the clinical setting to which the patient belonged, two levels were established depending on whether they received treatment in hospital or outpatient regimen. Respect pharmacological treatment, 4 levels were established according to the medication they were taking at the time of the evaluation: typical antipsychotics, atypical antipsychotics, typical and atypical antipsychotics, or other medications not related to psychotic illness.

### Assessment

#### Execution Tasks

*Cool* and *hot* EEFF tasks based on experimental paradigms of cognitive neuroscience were used, widely used in both, behavioral studies and functional neuroimaging studies with patients with brain damage, schizophrenia and healthy subjects (v.g., Allport et al., 1994; Frith et al., 1995; Baron-Cohen et al., 1997; Bechara et al., 1999; Menon et al., 2001; Asevedo et al., 2013; Thuaire et al., 2020). All tasks were programmed with the E-prime software (Schneider et al., 2002), which controls the presentation of the stimuli and the collection of the participants’ responses.

To obtain information about *cool* EEFF, 4 different tasks were designed: (a) Sternberg-type task, (b) 2-back task, (c) Letter-Number task, and (d) a computerized version of the Tower of Hanoi (THO). For the EEFF *hot*, 3 tasks were designed: (a) a computerized version of the Iowa Gambling Task, (b) a facial emotional expression recognition task, and (c) the Hinting task. Next, a more detailed description of each of the tasks and behavioral scales used will be made (see Table 2).

**TABLE 2:** Tasks to evaluate the components of the *cool* and *hot* executive functions and behavioral scales used in the study.

**Measure**	**Instrument**
*Cool components of the EEFF*	
Encoding/maintaining the information in the WM	Sternberg-type task (Sternberg, 1966)
Monitoring and updating information in the WM	2-Back Task (Fletcher, 2001)
Ability to change or alternate the mental set	Number-Letter task (Rogers and Monsell, 1995)
Planning	Computerized version of the Tower of Hanoi (Borys et al., 1982).
*Hot components of the EEFF*	
Decision-making under uncertainty	Computerized version of the Iowa Gambling Task (Bechara et al., 1994).
Recognition of the basic and complex expressions	Facial emotional expression recognition task (Baron-Cohen et al., 1997)
Theory of mind	Spanish version of the Hinting Task test (Gil et al., 2012)
*Psychotic symptoms*	
Negative symptoms	Scale for the Assessment of Negative Symptoms (SANS) (Andreasen, 1989)
Positive symptoms	Scale for the Assessment of positive Symptoms (SAPS) (Andreasen, 1984)
*Dysexecutive syndrome*	
Behavioral disorders of the frontal systems	Spanish version of the Frontal Systems Behavior Scale -FrSBe- (Pedrero et al., 2009)

*EEFF, executive function; WM, working memory.*

#### Cognitive or *Cool* EEFF Tasks

##### Sternberg-type task (Sternberg, 1966)

One of the most widely used paradigm to tests the processes of encoding/maintaining the information in the Working Memory (WM) is Sternberg item recognition task. These tasks consist of presenting the subject with a set of stimuli of variable amplitude for a short period of time, and then a single stimulus (the objective) is shown to indicate if said stimulus is one of those previously presented. In previous neuroimaging studies (e.g., Rypma et al., 1999; Rypma and D’Esposito, 1999; Siffredi et al., 2017), in which “letters” have been used as stimuli, it has been seen that if the subject must recognize only one letter, the left ventrolateral prefrontal cortex is activated, but if you must identify four or more letters, the dorsolateral prefrontal cortex is activated, so it has been suggested that the dorsolateral prefrontal cortex would be involved in those situations in which we must temporarily maintain information that exceeds the capacity of the “phonological loop,” (one of the components of specific modality of the WM). In other words, registering and maintaining three letters would depend exclusively on the WM phonological loop, but from that number of stimuli upwards, the participation of executive-type functions is required, specifically the WM Central Executive System, whose operation has been associated with dorsolateral PFC activity (Kruggel et al., 2000; Tirapu-Ustárroz and Muñoz-Céspedes, 2005; Altamura et al., 2007).

**FIGURE 2:**
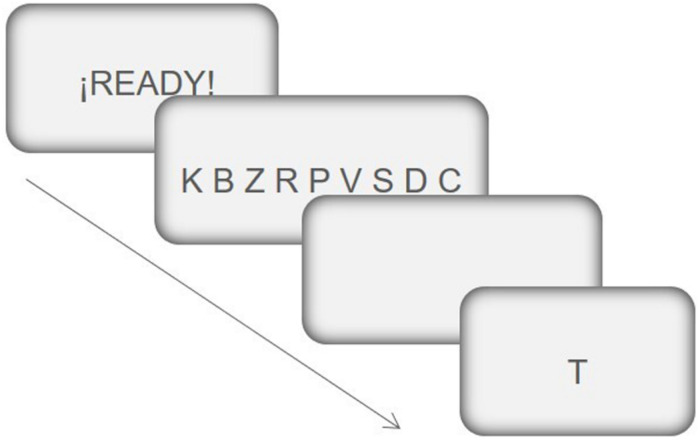
Example of the sequence of events in a trial of the Sternberg-type task. (1) First, a message appears for 1 s, alerting the subject of the imminent appearance of the information relevant to the task. (2) Once the alert message disappears, a row of 9 consonants appears for 9 s. (3) Next, there is a 5 s delay period, and lastly; (4) a single consonant appears in the center of the screen. The subject must indicate, by pressing a key, if said consonant was in the row of consonants previously presented. This consonant remains on the screen until the subject gives the answer. To answer “Yes” the subject has to press the “m” key and to answer “No” the “c” key on the computer keyboard. This figure has been adapted from the original task of Sternberg (1966).

In the task used in the present study, the participant is presented with a previous set of verbal stimuli (between three and nine letters), which remain on the screen for a time ranging from 3 to 9 s (according to the previous stimulus set amplitude). Then, after a delay of 500 ms, a single letter (target) is presented in the center of the screen and the participant must indicate (by pressing the corresponding key), whether said stimulus was present or not, in the previous stimulus set. The target remains on the screen until the answer, to go to the next trial the participant must press the space bar on the keyboard. All the consonants of the alphabet were used as stimuli (source: Times New Roman; size: 36) (see Figure 2).

The task has two conditions of stimuli load: (a) low load: in the previous stimuli set, between 3 and 5 letters appear; (b) high load: between 6 and 9 letters. Once the instructions are given to the participants verbally (which were also written on the computer screen), a block of 5 practice trials is presented, followed by the experimental block with a total of 56 trials (8 tests of each loading condition, which appear in random order). In 50% of the trials the target coincides with one of the letters presented in the previous stimulus set, and in the remaining 50% of trials it does not match. For each participant, the percentage of errors in each of the 2 stimuli load conditions is recorded.

##### 2-Back Task (Fletcher, 2001)

The n-back paradigm has been one of the most used to study the processes of monitoring and updating information in the WM. In this type of task, the subject is presented with a sequence of stimuli and must indicate which stimulus is identical to another presented *n* positions before, thus allowing the ability to actively maintain and regulate a limited amount of information relevant to the task to be evaluated (Pelegrina et al., 2015). Execution in these tasks has been associated with activity in the dorsolateral PFC, an area that incorporates specific computational mechanisms to monitor and manipulate cognitive representations (Callicott, 2000; Barbey et al., 2013).

**FIGURE 3:**
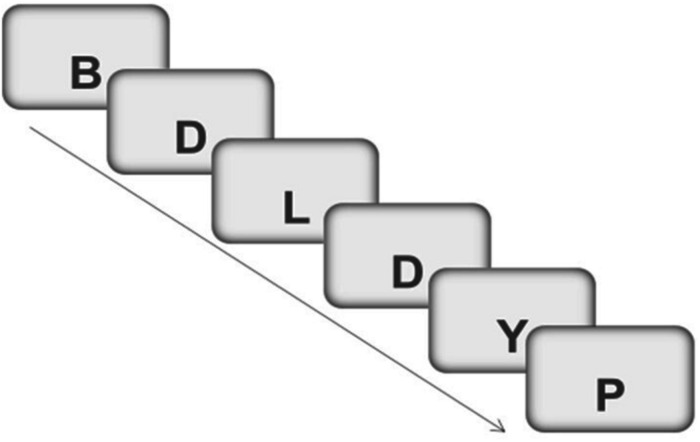
Example of a trials sequence in 2-back task. The subject must answer “YES” (by pressing the “1” key) when the letter “D” (target) appears a second time. In the rest of the trials (no-target) the subject must answer “NO” (by pressing the “2” key). This figure has been adapted from the original task of Robinson and Fuller (2004).

In the present study an adaptation of a level 2 n-back task developed by Robinson and Fuller (2004) was used. The verbal stimuli used in this task were also consonant. Specifically, the following 20 consonants were used: B, C, D, F, G, H, J, K, L, M, N, P, Q, R, S, T, V, W, Y, Z (source : Palatino Linotype, size: 30). Each letter was presented one by one on the screen for 500 ms, followed by a screen that remained blank for 3000 ms, so the participant has a maximum time of 3500 ms to respond. If the letter displayed on the screen corresponded to the one presented 2 positions before, you should press number 1 on the computer keyboard, if the letter did not correspond to the one presented two positions before, you should press number 2 (see Figure 3).

For each participant, the percentage of hits is recorded, understood as the percentage of “Yes” responses in the trials in which a target stimulus appears (a consonant that coincides with the one presented two positions before). The percentage of false alarms is also recorded (percentage of “Yes” responses in trials in which a target stimulus does not appear). From these two percentages and applying the parameters of the Signal Detection Theory - TDS-, the sensitivity index *d-prime* or *a-prime* can be calculated (Stanislaw and Todorov, 1999).

##### Number-Letter task (Allport et al., 1994; Rogers and Monsell, 1995)

This task is based on the task-switching paradigm, which evaluate the ability to change or alternate the mental set between a set of different responses (cognitive flexibility), depending on the demands of the situation. In this paradigm, the participant must quickly alternate between two or more types of tasks, which forces a continuous configuration and reconfiguration of the processes and operations necessary for their execution. In these tasks, an effect called “task-switching costs” (TSC) is observed, which indicates a lower speed or accuracy in the response of the subjects when they have to execute a change in the task or in the response criterion, in comparison with the performance achieved when they do not have to make such a change. Execution in this type of task has been related to activation in different brain areas, mainly the anterior cingulate cortex (ACC), the posterior parietal cortex (PPC), and the dorsolateral region of the PFC. Each area is related to a specific operation, so that the ACC and the PPC would act together to detect dissociable forms of conflict, at the response level (e.g., consistent or inconsistent) and at the stimulus level (e.g., relevant or irrelevant) respectively; while dorsolateral PFC would be required when the difficulty of the task increases and greater control is required (Sohn et al., 2000; Liston et al., 2006).

**FIGURE 4:**
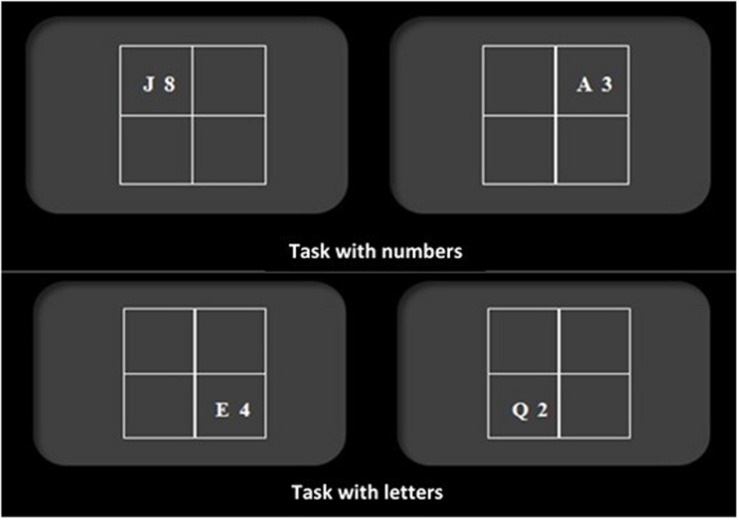
Examples of trials in Number-Letter task. If the stimulus appears in one of the two upper quadrants, the subject must respond according to the number, indicating whether it is odd or even. If the stimulus appears in one of the two lower quadrants, it must respond according to the letter, indicating whether it is a consonant or a vowel. This figure has been adapted from the original task of Rogers and Monsell (1995).

In the task used in the present study (adapted from Rogers and Monsell, 1995), the subject is presented with a number and a letter (e.g., “5G”) in one of the four quadrants of a matrix that appears in the center of the computer screen (see Figure 4). Subjects are told that when the number-letter pair appears in one of the two quadrants at the top, they will have to indicate whether the number is even or odd (by pressing the “m” key if it’s even and the “n” if it’s odd); but if it appears in one of the two quadrants at the bottom, they will have to indicate if the letter is a vowel or a consonant (by pressing the “z” key if it’s a vowel and the “x” key if it’s a consonant). The stimuli used in this task were: the consonants “G,” “K,” “M,” and “R”; the vowels “A,” “E,” “I,” and “U”; and the numbers from 2 to 9 (source: Times New Roman, size: 18).

The task consists of three trials blocks. In the first trials block (12 practice and 36 experimental), the letter-number always appears in one of the two quadrants at the top. In the second block, the letter-number always appears in the lower quadrants, and in the third block, the letter-number appears in both, the upper and lower quadrants.

For each participant, two TSC scores are obtained: The TSC score with reaction time (TSC^*TR*^) is obtained by subtracting the average reaction time obtained in the task change condition of block 3 and the average reaction time obtained in blocks 1 and 2. The TSC with errors (TSC^*E*^) is obtained by subtracting the percentage of errors obtained in the task change condition of block 3 and the percentage of average errors obtained in blocks 1 and 2.

##### Computerized version of the Tower of Hanoi (Borys et al., 1982)

This task is based on the so-called “tower tests” (Soprano, 2003), which allow evaluating the planning processes that involve the preparation and representation of ordered sequences of actions to achieve specific objectives (Greenwood et al., 2011). The execution of these tasks has been mainly associated with the activation of the dorsolateral PFC, both, with its right and left sides, but each with a different specificity; the right dorsolateral area would be required in the construction of the plan to solve the planning problem, while the left area would be involved in the control processes, supervising the execution of the plan.

**FIGURE 5:**
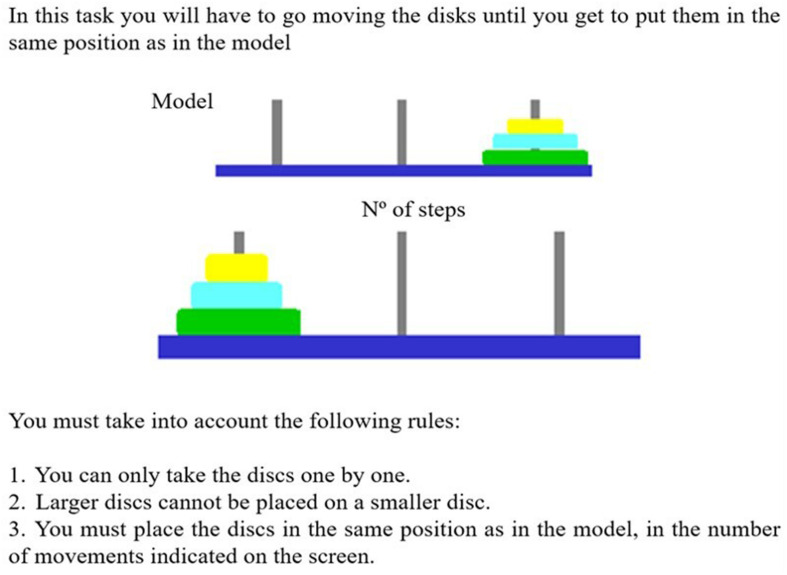
Instructions were presented to subjects in the computerized version of the Tower of Hanoi. This figure has been adapted from the original task of Groot (2004).

In the present study, an adaptation of the Tower of Hanoi by Groot (2004) was used. A total of 10 trials with increasing difficulty are presented in this task. Each trial consists of the presentation of two towers, one presented at the top of the screen that serves as a model and the other presented at the bottom, which is the one that the participant can manipulate. The task is to replicate the model by following a specified number of steps. To carry out this task, the participant, using the mouse, must manipulate the blocks of different sizes and colors until the correct sequence is achieved in the number of steps required (see Figure 5).

The task has two planning conditions according to the difficulty: (a) short planning in which less than 5 movements are required to complete the model; and, (b) long planning, in which more than 5 movements are required to complete the model. Both, the number of errors (incorrect movements) and the average latency time between movements are recorded.

#### Socio-Emotional or *Hot* EEFF Tasks

##### Computerized version of the Iowa Gambling Task (Bechara et al., 1994)

This task is one of the most widely used to study decision-making processes and was originally developed to simulate real-life decisions in terms of uncertainty, reward, and punishment. The neuroanatomical area associated with decision-making in situations of uncertainty is the orbitofrontal region of the PFC (Bechara et al., 1999).

In the present study, an adaptation of the Iowa Gambling Task carried out by Patterson et al. (2002) was used. A total of 100 trials are presented in this task. Each trial consists of the presentation on the screen of 4 decks of cards, each with a figure in the middle (diamond, circle, star, and square). Participants are instructed that the game consists of choosing cards from any of the four decks and that the objective is to accumulate as many points as possible.

**FIGURE 6:**
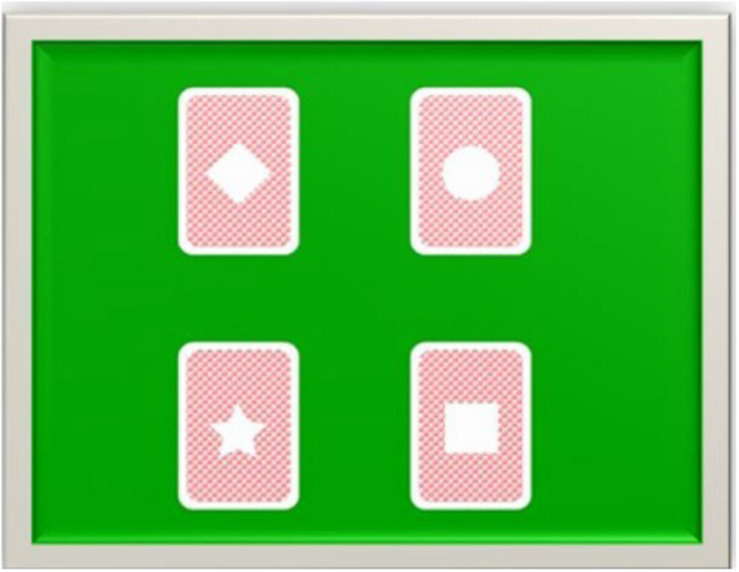
Stimuli used in the computerized version of the Iowa Gambling Task. In each trial, the subject must pick up a card from one of the four decks, pressing the corresponding key. Each time the subject makes a choice, the gain or loss associated with the choice they just made appears on the screen, as well as the number of points accumulated. This figure has been adapted from the original task of Patterson et al. (2002).

The four decks of cards can be divided into “disadvantageous” (star and diamond) and “advantageous” (circle and square). “Disadvantageous” decks provide high rewards (200 points for each choice) and high penalties: each cycle of 10 choices contains 2 variable penalties of −310 or −2.150 points for the star deck, and 4 penalties of −310 or −465 points for the diamond deck. “Advantageous” decks contain minor rewards (150 points in each election), and minor penalties: in each cycle of 10 elections, the circle deck contains a penalty of −1.000 points and the square deck four penalties of −125 or −145 points (see Figure 6).

After each choice, the participant is shown the number of points he has earned or lost, and the total points accumulated. Participants’ performance is evaluated by calculating the “net score,” that is, the number of cards selected from the “advantageous” decks minus the number of cards selected from the “disadvantageous” decks.

There are some differences between the present adaptation and the original task of Bechara et al. (1994). In our task the participants accumulate points and not game money. Additionally, our participants start the game with 0 points, the original task with $ 2,000. And finally, we use higher profit and loss scores: in our task, in each cycle of 10 choices of the “disadvantageous” decks, it is possible to earn 2,800 points and lose 4,010; and in the “advantageous,” earn 2,250 points and lose 1,345. In the original task, in “disadvantageous” decks it is possible to win $ 1,000 and lose $ 1,250, and in “advantageous,” it is possible to win $ 500 and lose $ 250.

##### Facial emotional expression recognition task (Baron-Cohen et al., 1997)

This task was designed with the purpose of evaluating the recognition of the basic and complex emotional expressions of the face, it assumes that for an adequate ToM, the recognition of secondary emotional states is required. Among the brain structures involved, the temporo-occipital cortex stands out, especially the fusiform gyrus, the orbitofrontal region of the PFC and the right parietal area, the amygdala, and the basal ganglia (Haxby et al., 2000).

In the present study, we have adapted and computerized the original task proposed by Baron-Cohen et al. (1997). The task consists of 3 blocks of 20 trials each, in each block, 20 black and white photographs of a model showing different emotional facial expressions: 10 basic and 10 complex emotions. The basic emotional expressions used were: happy, sad, angry, afraid, surprised, disgust, and distress. Just like in the original study, surprise, happy, and angry were repeated using new poses to form the set of 10 basic emotions. As for the complex emotional expressions: scheming, guilt, thoughtful, admiring, quizzical, flirting, bored, interested, and arrogant were used. Repeating the emotion of “interested” with a new pose to complete the 10 complex emotions. Each photograph was presented in the center of the screen accompanied by two words, one in the lower right and the other in the lower left of the photograph. Only one of the words describes the correct emotion and the other is used as a distractor.

The participant’s task is to choose the word that they think best describes the emotion that the model in the photograph is expressing. Each trial begins with the presentation of a central fixation point (+) for 1 s, and immediately afterwards the photograph appears along with the two words, which remain on the screen until the participant’s response. The error rate and reaction time were recorded for each participant, for both, basic and complex emotions.

##### Spanish version of the Hinting Task test (Gil et al., 2012)

This task was designed with the purpose of evaluating the capacity of mentalization or ToM in patients with schizophrenia. Specifically, it assesses the understanding of hints, ironies, or false beliefs (Corcoran et al., 1995). Execution in this type of tasks has been mainly associated with the medial region of the PFC and the posterior part of the ACC (Amodio and Frith, 2006).

The task used in the present study includes ten short stories that the evaluator can read to the participants as many times as necessary in order to ensure a correct understanding of them and reduce the interferences of the possible deterioration in memory or verbal comprehension. In all stories two characters appear and, at the end of each story, one of the characters drops a clear hint to the other character. The participant is asked what he thinks the character in the story really meant by the comment he made. Each story provides a series of criteria or accepted responses to guide scoring. If the person responds correctly, that is, according to these criteria responses, they are scored with a 2; if not, additional information from the story is added to make the hint even clearer. If this time it answers correctly, according to the criterion answers, it is scored with a 1, if it does not answer correctly it is scored with a 0. From this task a direct score is obtained that goes from 0 to 20 points. The higher the score the better the capacity for mentalization or ToM.

#### Behavioral Scales

##### Scale for the Assessment of Negative Symptoms -SANS- (Andreasen, 1989)

This scale was used to obtain a measure of the severity of NSs in the patient’s group. This scale is made up of a total of 30 items grouped into five subscales: affective flattening, alogia, avolition-apathy, anhedonia-asociality and Attentional impairment. Each item evaluates behaviors usually associated with NSs and is rated on a scale of 0 (not present) to 5 (severe). A score between 0 and 150 can be obtained. Higher scores indicate a greater presence and severity of NSs. These scores were transformed into a percentage, where we have taken a higher percentage compared to the Scale for the Assessment of Positive Symptoms (SAPS) as equivalent to a greater prevalence of NSs.

Approximately 30 min are required for its application and it is recommended that the scale be completed by trained evaluators based on a standard clinical interview, also taking into account the behaviors observed during the interview, and the information from the patient’s medical history.

Regarding test-retest reliability, the correlation index (CI) was 0.80, regarding its validity, the correlations of the SANS with the Negative Subscale of the Scale of Positive and Negative Symptoms (PANNS) was 0.88; and with the NSs of the Brief Scale of Psychiatric Symptoms (BPRS) it was 0.85 (Peralta and Cuesta, 1999).

##### Scale for the Assessment of Positive Symptoms -SAPS- (Andreasen, 1984)

To select the 33 psychotic patients with a predominance of NSs from the largest sample of 66 patients, it was also necessary to administer a scale that provided information on the presence and severity of positive symptoms. The SAPS scale is made up of 34 items grouped into four subscales: hallucinations, delusions, extravagant or strange behavior, and formal thought disorder. Each item evaluates behaviors usually associated with positive symptoms and is rated on a scale of 0 (not present) to 5 (severe). A score between 0 and 170 can be obtained. Higher scores indicate a greater presence and severity of positive symptoms. This score was transformed into a percentage to compare it with the score of the Negative Symptom Assessment Scale (SANS) and establish the symptomatic predominance. An approximate time of 30 min is required for its application, and it must be administered by a trained evaluator. Regarding its test–retest reliability, the correlation index (CI) was 0.73, and regarding its validity, the correlations with the Positive Subscale of the PANNS were 0.91; and with the positive symptoms of BPRS it was 0.89 (Peralta and Cuesta, 1999).

##### Spanish version of the Frontal Systems Behavior Scale -FrSBe- (Grace and Malloy, 2001; Pedrero-Pérez et al., 2009)

This scale provides a measure of the behavioral disorders associated with the three syndromes of frontal origin: anterior cingulate syndrome (apathy), orbitofrontal syndrome (disinhibition) and dorsolateral syndrome (executive dysfunction). It allows obtaining a measure of behavioral changes considering the temporal dimension, since they allow comparison of behavior before and after injury or alteration. There are two versions: one self-reported and the other that must be completed by a family member or the patient’s caregiver. Only the self-reported form was used in the present study. It consists of a total of 46 items grouped into three independent subscales: apathy (14 items), disinhibition (15 items) and executive dysfunction (17 items). In the present study, only the executive dysfunction subscale score was used, which provides a measure of the behavioral disturbances associated with the dorsolateral prefrontal syndrome or dysexecutive syndrome. Items on this subscale are scored on a 5-point Likert-type scale (1 = almost never, 2 = rarely, 3 = sometimes, 4 = frequently, 5 = almost always). The FrSBe has shown adequate construct validity to evaluate the different clinical syndromes of frontal origin (Pedrero-Pérez et al., 2009; Caracuel et al., 2012).

### Procedure

Regarding the neuropsychological evaluation of the *hot* and *cold* EEFF, both, for the patients and the control group, these were carried out by two single researchers, one of the researchers always carried out the evaluation and the second researcher supervised mentioned evaluation. A blind trial was not carried out, however, as they are computerized tasks, provide a series of advantages that allow minimizing the influence of the researcher on the participant’s performance, such as the possibility of obtaining more precise and exact scores, reducing errors in data collection since the participants respond directly to the computer, allowing to obtain more precise times and hits. To administer all the tasks in the group of patients, each of them required two individual sessions of approximately 50 min in duration, each with the necessary breaks they required, to promote their motivation and active participation in carrying out tasks. In the case of the control group participants, the majority required a single session of approximately 60 min, with the necessary breaks required. The evaluation sessions were carried out individually in a quiet room using a laptop.

Respect to the evaluation of the clinical symptoms of the patients, the SANS and SAPS scales were administered by the reference psychiatrists or clinical psychologists. For the self-reported version of the FrSBe “Executive dysfunction” subscale, the patient was given the option of completing it alone (in the presence of the investigator) or with the help of the investigator, always trying to ensure maximum understanding of patients’ questions.

To select psychotic patients with a predominance of NSs, the following procedure was followed. Once the patient’s reference psychiatrists or clinical psychologists completed the SANS and SAPS scales for each patient, the total scores on each scale were calculated. Each score was then transformed into a percentage. For the SANS scale, the percentage was calculated based on the maximum score that can be obtained on this scale (150), following the same procedure for the SAPS scale (maximum score = 170). Finally, those patients who presented more negative (*M* = 39.9, *DT* = 25,40) than positive symptoms (*M* = 15.7, *DT* = 15,05) were selected.

### Statistical Analysis

An exploratory analysis and cleaning of the data was carried out. Two cases were identified with missing data in two response variables that were imputed to the mean of the group they belong to. Outlier data were identified, however, no procedure was performed, because they were consistent with the nature of the evaluated. Frequency and percentage measures were estimated for the characterization of the sociodemographic and clinical variables. The analysis of *X*^2^ was carried out between the groups and gender and level of education. The difference between patients and control in the sociodemographic variable, age, was estimated with the U de Mann–Whitney test. Measures of central tendency (M, Mean) and dispersion (SD, Standard Deviation) of the direct scores were estimated for informational purposes. Next, the direct scores were transformed to *Z* scores, which allows standardization and comparison with previous works. Two multivariate analysis models (Manova) were run for each group of measures of EEFF. The first model was made up of four *cool* EEFF tasks × two groups (4x2). The second model contrasted three *hot* EEFF × two groups (3x2). Assumptions testing for hypothesis testing was carried out using standardized residuals for normality in both groups. The assumption of the equality of covariances was estimated with Box’s Test = 2079, *p* = 0.000. Therefore, the multivariate test was Pillai’s Trace. The analysis of comparisons of means was corrected Bonferroni. In the comparisons that showed significant differences, the confidence interval (95% CI) was reported. The effect size estimated with partial eta squared ($\eta_{\text{p}}^{2}$), the following values are considered: <0.01 small, 0.06, moderate, >0.14 strong (Cohen, 1988; Ellis, 2010). The data treatment was through SPSS v.23.0. *Post hoc* statistical power (1-β) was calculated with G^∗^Power software (Faul et al., 2007).

Regarding the possible relationship of the clinical variables in the execution of the *cool* and *hot* EEFF tasks, the following analyzes were carried out: the influence of the variables duration of illness and clinical setting was estimated with the t student test for independent samples, for the variable pharmacological treatment an parametric ANOVA one way was carry out. Tukey’s Test for Post-Hoc analysis was carried out. Regarding our second objective, two correlation analysis was carried out. On the one hand, between the severity of the NSs and the EEFF tasks (*cool* and *hot*); and, on the other hand, behavioral changes (self-report) and EEFF tasks (*cool* and *hot*), Pearson’s r correlation coefficient was calculated, both, for the total score of the SANS and EEFF tasks, as well as for the score of the subscale “Executive dysfunction” of the FrSBE and EEFF tasks, respectively. Interpreted with reference to 0.05 significance levels and Bonferroni correction.

## Results

The final sample consisted of *n* = 66 participants (Range of age min = 20 – max = 60), both genders: male (*n* = 49, 74.2%, *M_age_* = 43.6, *SD* = 11.0), female (*n* = 17, 25.8%, *M_*age*_* = 44.2, *SD* = 11.0); 33 psychotic patients (paranoid schizophrenia *n* = 31 or schizoaffective disorder *n* = 2), and 33 participants in the control group. The sociodemographic and clinical characteristics are observed in Table 3. No differences were found between patients vs. controls in age, U(*N_*pati*__*e*__*nts*_* = 33, *N*_*controls*_) = 542.0, *z* = −0.03, *p* = 0.974, gender, [*X^2^(1)* = 0.79, *p* = 0.778], or years of education [*X^2^(2)* = 0.83, *p* = 0.959].

**TABLE 3:** Sociodemographic and clinical variables of the patient and control group.

**Variables**	**Patients *n* = 33**	**Controls *n* = 33**	**All *n* = 66**
	***f* (%)**	***f* (%)**	**(*f*, %)**
Sociodemographic			
Age_*years old*_ M(±)	44.3 ± 9.0	44.1 ± 12.7	43.7 ± 10.9
Gender			
Male	24(72.7)	25(75.8)	49(74.2)
Female	9(27.3)	8(24.2)	17(25.8)
Schooling_(__*years)*_			
Basic (<6)	17(51.5)	16(48.5)	33(50.0)
Medium (7 and 12)	9(27.3)	10(30.3)	19(28.8)
High (>12)	7(21.2)	7(21.2)	14(21.1)
Clinical			
Years of evolution of the disease			
Short	16(48.5)	–	–
Long	17(51.5)	–	–
Clinical treatment device			
In-hospital	18(54.5)	–	–
Outpatient	15(45.5)	–	–
Pharmacological treatment			
Typical antipsychotics	4(12.1)	–	–
Atypical antipsychotics	18(54.5)	–	–
Typical and atypical antipsychotics	3(9.1)	–	–
Other medications	8(24.2)	–	–

Descriptive data (direct scores and *Z* scores) between patients and controls are shown in Table 4. The multivariate-MANOVA analysis indicated an effect in the interaction between the performance of the tasks of the *cool* EEFF × groups, Pillai’s Trace *V* = 0.434, *F* = (9,56) = 4.06, *p* = 0.001, $\eta_{\text{p}}^{2}$ = 0.434, 1-β = 0.99. Similarly, an effect was observed in the interaction between the performance of the tasks of *hot* EEFF × groups Pillai’s Trace *V* = 0.434, *F* = (6,59) = 21.13, *p* = 0.001, $\eta_{\text{p}}^{2}$ = 0.682, 1-β = 1.0 (see Table 4).

**TABLE 4:** Descriptive of direct score, transformed score (Z) and multivariate Manova patients vs. controls.

**Executive functions**	**Direct Score**		**Score *Z***		***MS***	***F***	***$\eta_{\text{p}}^{2}$***
	**Patients**	**Control**	**Patients**	**Control**			
***Cool tasks***							
Sternberg-type task							
Low load_(% *Errors*)_	10.1(10.7)	3.0(7.3)	0.35(1.0)	−0.35(0.7)	8.59	9.45**	0.13
High load_(% *Errors*)_	21.7(12.3)	12.8(9.4)	0.35(1.0)	−0.36(0.7)	9.51	10.7**	0.15
2-Back task							
a-prime index_(accuracy)_	0.7(0.2)	0.8(0.1)	−0.41(1.0)	0.42(0.5)	11.1	13.0***	0.17
Number-letter task							
TSC_*RT(sec)*_	1.6(1.2)	0.62 (0.2)	0.36(1.2)	−0.47(0.2)	11.2	17.5***	0.22
TSC_(errors)_	9.9(19.4)	0.2(1.6)	0.34(1.3)	−0.34(0.1)	7.51	8.24**	0.11
Tower of Hanoi (planning)							
Short_(Errors)_	0.3(0.6)	0.3(0.5)	0.03(1.1)	−0.03(0.9)	0.04	0.04	0.00
Long_(Errors)_	1.8(1.2)	1.7(1.3)	0.04(0.9)	−0.04(1.0)	0.00	0.00	0.00
Short_(Latency, sec__*onds*__)_	31.1 (21.6)	21.7(11.9)	0.26(1.2)	−0.26(0.6)	3.48	3.80	0.05
Long (_Latency, sec__*onds*__)_	75.9 (44.6)	57.1(34.3)	0.23(1.0)	−0.23(0.8)	1.17	2.07	0.03
***Hot tasks***							
Iowa gambling Task							
Net score	0.9(4.1)	1.3(3.6)	−0.06(1.0)	0.06(0.9)	0.20	0.20	0.00
Facial emotional expression recognition task							
Basic emotions_(__%E__*rrors)*_	13.8(11.1)	18.0(6.1)	−0.23(1.2)	0.23(0.6)	3.59	3.75	0.05
Complex emotions_(__%E__*rrors)*_	31.1(10.8)	26.2(8.7)	0.24(1.0)	−0.24(0.8)	3.94	4.13	0.06
Basic emotions_RT(sec)_	5.7(2.5)	3.1(0.9)	0.56(1.1)	−0.56(0.3)	20.9	30.5***	0.32
Complex emotions_*RT(sec)*_	7.2(3.9)	3.3(1.1)	0.55(1.1)	−0.55(0.3)	20.1	28.8***	0.31
Hinting Task							
Direct score	14.0(4.3)	18.8(1.3)	−0.60(1.0)	0.60(0.3)	24.1	37.8***	0.37

TCS, task-switching costs; RT, response time; MS, mean square. **p < 0.01, ***p < 0.001.

### *Cool* EEFF Tasks

A main effect was found in the task of *coding/maintaining information in the WM* (Sternberg-type task), both, when had to code and maintain between 3 and 5 letters, low load condition *F* = (1,64) = 9.45, *p* = 0.003, $\eta_{\text{p}}^{2}$ = 0.132, 1-β = 0.86; as in the condition in which they had to code and keep between 6 and 9 letters, high load condition *F* = (1,64) = 10.75, *p* = 0.002, $\eta_{\text{p}}^{2}$ = 0.149, 1-β = 0.89. Similarly, in the task of *updating the information in the WM* (task 2-back) a main effect was also found *F* = (1,64) = 13.05, *p* = 0.001, $\eta_{\text{p}}^{2}$ = 0.174, 1-β = 0.94.

Regarding the ability to *change the mental set* (task number-letter) a main effect of TSC was found, both, with reaction times *F* = (1,64) = 17.51, *p* = 0.001, $\eta_{\text{p}}^{2}$ = 0.220, 1-β = 0.98; and with the percentage of errors *F* = (1,64) = 8.24, *p* = 0.006, $\eta_{\text{p}}^{2}$ = 0.117, 1-β = 0.80. Regarding the planning task (Tower of Hanoi), no main effects were found (see Table 4).

### *Hot* EEFF Tasks

Respect for the three *hot* EEFF tasks, we only found a main effect in two of the tasks used (see Table 4).

Regarding *decision-making in situations of uncertainty* (Iowa Gambling task), no main effect was found. Respect to *facial emotional expression recognition task*, only was found a main effect in the measurement of reaction time (RT), both, in basic *F* = (1,64) = 30.52, *p* < 0.001, $\eta_{\text{p}}^{2}$ = 0.323, 1-β = 1.0, and complex emotions *F* = (1,64) = 28.84, *p* < 0.001, $\eta_{\text{p}}^{2}$ = 0.311, 1-β = 1.0. Finally, the performance in the ToM task (Hinting Task) showed a significant effect *F* = (1,64) = 37.82, *p* < 0.001, $\eta_{\text{p}}^{2}$ = 0.371, 1-β = 1.0.

### Differences Between Patients and Control Subjects in EEFF Tasks

**FIGURE 7:**
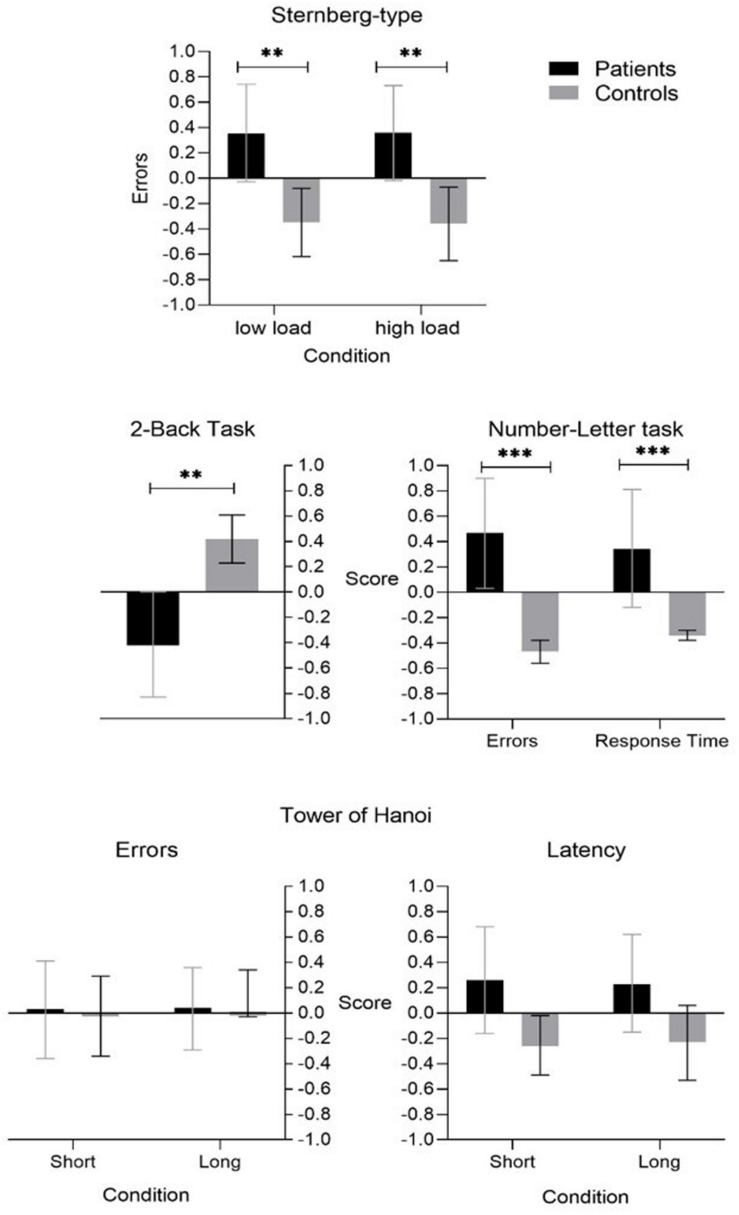
Cool Executive Functions tasks. **p < 0.01, ***p < 0.001.

As we expect, in the comparison of the marginal means it was observed that the control group showed better performance in the *cool* executive functions (see Figure 7). Respect to *executive components of the Working Memory* (WM), In the task of *coding/maintaining information in the WM* (Sternberg-type task) significant differences were found, the patients had a higher percentage of errors, both, when they had to code and maintain between 3 and 5 letters, low load condition (*p* = 0.003, 95%CI [0.25,1.21]); as in the condition in which they had to code and keep between 6 and 9 letters, high load condition (*p* = 0.002, 95%CI [0.30, 1.24]). In the task of *updating the information in the WM* (task 2-back) a significant difference was also found (*p* = 0.001, 95%CI [−1.29,−0.37]), the patient group had a lower performance than the control group, having a lower a-prime sensitivity index, which would correspond to a lower sensitivity to detect stimuli. Regarding the ability to *change the mental set* (task number-letter) significant differences were found, the group of patients compared to the subjects in the control group, showed a greater effect of TSC, both, with reaction times (*p* < 0.001, 95%CI [0.43.29,1.24]), and with the percentage of errors (*p* = 0.006, 95%CI [0.20,1.16]).

**FIGURE 8:**
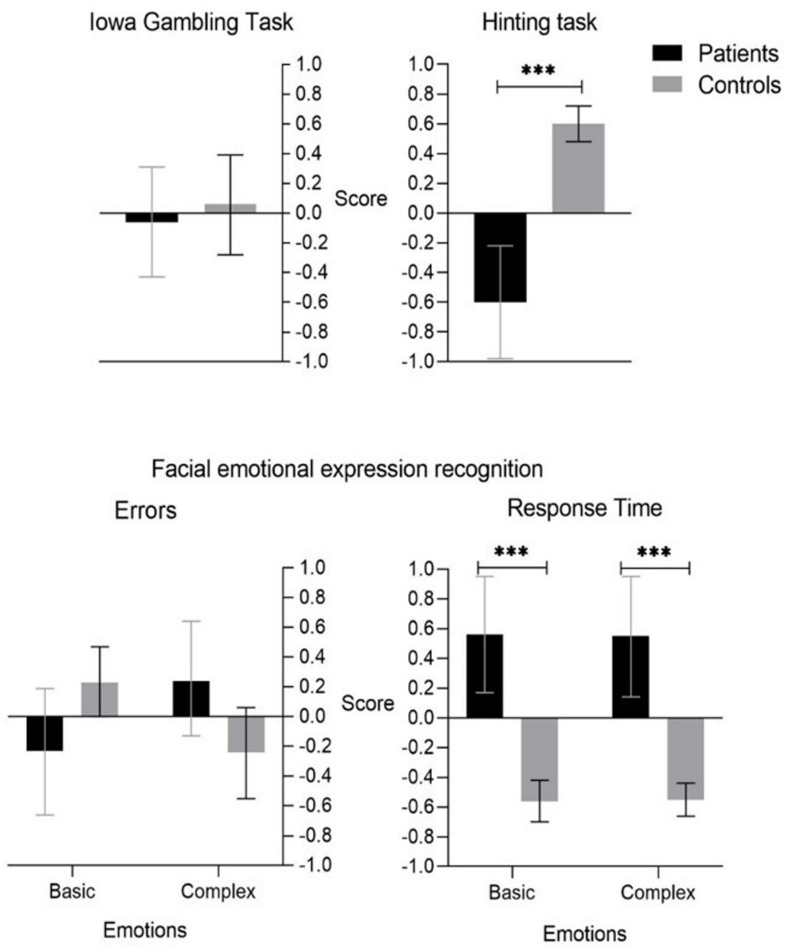
Hot Executive Functions tasks. **p < 0.01, ***p < 0.001.

Concerning the 3 *hot* EEFF tasks (see Figure 8) we only found significant differences between patients and controls in two of the tasks used (recognition of emotional facial expressions and ToM).

Respecting *decision-making in situations of uncertainty* (Iowa Gambling task), both, control subjects and patients made a greater number of advantageous than non-disadvantageous choices, and although the net score of the patient group (0.9) was somewhat lower than the control group (1.3) no main effects were found.

Respect to *facial emotional expression recognition task*, the patients showed a lower performance than the control group subjects, although no significant differences were found in the percentage of errors in both, basic and complex emotions, significant differences were found in the measurement of reaction time (RT) where patients had significantly higher RTs than controls, both, in basic (*p* < 0.001, 95%CI [0.72,1.53]), and complex emotions (*p* < 0.001, 95%CI [0.69,1.51]).

Finally, significant differences were found in the ToM task, the patient group obtained a significantly lower score than the control group (*p* < 0.001, 95%CI [−1.60,−0.81]).

### Relationship of Clinical Variables on the Execution of *Cool* and *Hot* EEFF Tasks in the Patient Group

Concerning the variable duration of the disease, no differences were found between patients with less than 11 years and patients with more than 11 years of duration. Respect to the clinical setting to which the patient belonged (treatment in hospital or outpatient regimen), differences were only observed in the Tower of Hanoi task, in long planning errors {*t*(31) = −2.20, *p* = 0.035, 95%CI [−1.32, −0.05]}, and long planning latency {*t*(31) = −2.93, *p* = 0.006, 95%CI [−1.71, −0.30]}, with poor performance in patients with hospital regimen. Regarding to pharmacological treatment, significant differences were found in the Tower of Hanoi task in long planning errors *F* = (3,29) = 4.85, *p* < 0.007, $\eta_{\text{p}}^{2}$ = 0.334, Tukey’s test for *post hoc* analysis test found differences between patients treated with typical antipsychotics, compared to atypical and others not related to psychotic illness, these being the ones that showed lower performance.

### Correlations Between Negative Symptoms, Behavior Dysexecutive and Performance in *Cool* and *Hot* EEFF Tasks in Patients

Correlation analysis showed that SANS scores were related to short planning performance in errors (*r* = 0.35, *p* = 0.046, 95% CI [0.65,0.02]) and latency in the long condition (*r* = 0.35, *p* = 0.039, 95% CI [0.62,0.02]). On the other hand, the score of the subscale “Executive dysfunction” of the FrSBe, were related to the Tower of Hanoi task in the condition of latency in short planning (*r* = 0.48, *p* = 0.005, 95% CI [0.70,0.16]) (see Table 5). However, after applying the respective correction (*p* > 0.001 with Bonferroni correction) these findings have not survived, finding a non-significant correlation, both, for the SANS score and for the score of the subscale “Executive dysfunction” with all executive functions tasks.

**TABLE 5:** Correlations coefficients between predominance symptoms and behavior dysexecutive (*r* Pearson).

**Task**	**SANS**	**FRSB**
***Cool tasks***		
Sternberg-type task		
Low load_(% *Errors*)_	−0.10	0.11
High load_(% *Errors*)_	−0.03	0.23
2-Back task		
a-prime index_(__*accuracy*__)_	−0.03	−0.20
Number-letter task		
TSC_*RT(sec)*_	0.21	−0.03
TSC_(errors)_	−0.19	−0.15
Tower of hanoi (planning)		
Short_(Errors)_	0.35*	0.20
Long_(Errors)_	0.33	−0.08
Short_(Latency, sec__*onds*__)_	0.26	0.48**
Long (_Latency, sec *onds*_)	0.36*	0.12
***Hot tasks***		
Iowa gambling Task		
Net score	0.07	−0.05
Facial emotional expression recognition task		
Basic emotions_(__%E__*rrors)*_	0.14	16
Complex emotions_(__%E__*rrors)*_	−0.08	0.32
Basic emotions_RT(sec)_	−0.00	0.15
Complex emotions_*RT(sec)*_	−0.03	15
Hinting Task		
Direct score	−0.08	−0.04

*p < 0.05, **p < 0.01 without Bonferroni correction.

## Discussion

The present study had two objectives. On one hand, the specific deficits in a series of c*ool* and *hot* Executive Functions tasks, in a group of patients with a predominance of negative schizophrenic symptoms, compared to a control group were analyzed; likewise, the influence of clinical variables (duration of the disease, clinical setting and pharmacological treatment), was also explored in the performance of tasks.

On the other hand, we studied the degree of correlation between NSs (measured through the Scale for the Evaluation of Negative Symptoms -SANS-) and the performance in *cool* and *hot* EEFF tasks, as well as its relationship with behavioral disturbances related with dysexecutive syndrome (measured through the Executive Dysfunction subscale of the Frontal Systems Behavior Scale -FrSBe-).

### Alterations in *Cool* and *Hot* EEFF Tasks

As expected, a significantly lower performance was found by the group of patients compared to the control group in all the *cool* EEFF tasks.

As for the *working memory (WM) deficiencies* found in our study, these are consistent with previous literature (Carter et al., 1996; Menon et al., 2001). WM refers to the system that temporarily maintains and manipulates information, it is mainly composed of three different components: the phonological loop (temporary storage of verbal information), the visuospatial sketchpad (temporary storage of visual information) and the central executive system, which manipulates the information of the two previous components, activating itself in novel situations that require control and supervision. This executive system has two main functions: the encoding/maintenance of information when the capacity of the loop and the visuospatial agenda is saturated, and the capacity to update information.

In our study, patients compared to the control group, presented a higher percentage of errors in the task of *coding/maintaining information in WM* (Sternberg-type task), and they have obtained a worse execution in the task of *updating the information in the WM* (2-back task). In this sense, studies such as those by Hartman et al. (2003) emphasize the involvement of the information coding/maintenance process in patients with schizophrenia, where the difficulties would be or the perceptual inability to select the relevant information requiring more time of exposure to the stimulus, or not adequately deploying attention to the relevant characteristic in an efficient way, which would hinder the coding process.

However, our patients not only presented difficulties in the process of coding/maintaining the information, they also presented difficulties in updating the contents of working memory, a process that requires the manipulation, monitoring and temporal reordering of the information. Therefore, our results could suggest the existence of involvement in more than one WM process. Along these same lines, authors such as Lee and Park (2005) suggest that imprecise coding by itself would not explain the WM deficits in these patients, in fact, for authors such as D’Esposito et al. (1998) the Coding/maintenance and updating are not completely dissociable processes, since coding may require strategic processing with increasing load, and some degree of manipulation and updating may be required to respond to a task. So, a deficit might be suggested in these patients broader than that reported by Hartman.

Regarding the ability to *change or alternate the mental set*, the difficulties in this aspect, have been related to perseveration problems, and with the difficulty that patients have to disengage attention (Waltz, 2017). In our study, patients compared to the control group had a higher cost of changing the mental set, both, in errors and in reaction time (RT), being in RT where a larger effect size or a greater difference between the two groups was found. Authors such as Meiran et al. (2000) using a similar task-switching paradigm to ours, have obtained similar results, attributing the high response latencies in these patients to the deficits in WM. For these authors, the trials of change require both, maintaining and updating the information according to the given key, in our case, according to the position of the stimulus (upper vs. lower quadrants), where patients would present a forgetfulness of the key to remember and the meaning of the responses and, therefore, they need to acquire them again in each trial delaying their execution.

On the planning component, the previous literature presents contradictory results. Some studies of schizophrenic patients point to a marked deterioration in planning ability and a slowdown in action (Greenwood et al., 2011), while others, found no significant differences with controls (Asevedo et al., 2013). In our study, the performance of the patients did not differ from that of the controls, although a greater slowing down of the patients was found for both, the short planning and long planning conditions, these differences were not significant. A possible explanation for these results is that, like the Asevedo et al. (2013) study, we have used a tower-type task (Hanoi Tower). Although these tasks have the advantage that they can be designed to test different skill levels, for some authors as Morris et al. (2005) these task are somewhat removed from planning “in the real world” where more open solutions and more flexible judgments are require. Therefore in patients with schizophrenia, especially in patients with NSs and marked poverty psychomotor, difficulties of a poor functioning in daily life could be masked.

Concerning the *hot* EEFF, in our study the patients showed an altered performance both, in the task of *recognition of facial emotional expressions* and in that of *ToM*.

The ability to recognize emotions through facial expressions plays a fundamental role in social interactions and communication, however, in our data, although patients make more errors in the recognition of complex expressions, this difference was not significant, showing more a slowdown in the recognition of emotions than a difficulty in discriminating them. This difficulty that patients present in the adequate or timely recognition of emotions can influence the ability to infer the mental states and intentions of others or ToM. In our study, patients reported a significantly worse performance in recognizing the intentions of others, these difficulties have been directly related to NSs such as affective flattening and asociality (Frith, 1992; Rodríguez et al., 2011). Our results, therefore, are consistent with those reported by the previous literature, in which a deterioration of these functions has been found in patients with schizophrenia (Browne et al., 2016).

Regarding the *clinical variables* analyzed in the present study (duration of illness, clinical setting to which the patient belonged – treatment in hospital or outpatient regimen – and pharmacological treatment), only significant differences were found in the planning task depending on the *clinical setting in the who receive the treatment* (in-hospital vs. outpatient), and depending on *pharmacological treatment* the patients were taking at the time of the evaluation.

Relate to the *clinical setting*, patients who regularly attend or live in the hospital (in-hospital), presented better results than outpatients, both, with precision and speed measurements in the long planning task. This result could be explained by the fact that the in-hospital patients included in our study are those who attend the day hospital and the therapeutic community, two clinical devices that allow intensive and comprehensive psychosocial treatment, and where adherence to pharmacological treatment is cared for.

About *pharmacological treatment*, likewise, only the influence of this variable was observed in the planning task (although only with the percentage of errors in the long planning condition), where patients treated with typical antipsychotics made more errors than those treated with atypical antipsychotics and other medication. In this sense, various studies have reported benefits on cognitive function and better performance on neurocognitive tasks in those patients who are treated with atypical vs. typical antipsychotics (e.g., Harvey and Keefe, 2001; Müller et al., 2005; Krakowski and Czobor, 2011). However, our result regarding the treatment variable, should be taken with caution for various reasons, first, we made a selection of patients by those who were taking typical, atypical and other medication not related to mental illness, but we did not perform a differentiation by calculating an estimate based on an average chlorpromazine equivalents (Gardner et al., 2010; Ballesteros et al., 2018). On the other hand, when assigning the patients to the different groups according to the type of pharmacological treatment, these are unbalanced, with the group of patients receiving atypical antipsychotics being much larger (*N* = 17), than the group of patients receiving antipsychotic treatment, typical (*N* = 4).

### Negative Symptoms, Dysexecutive Syndrome and Execution in EEFF Tasks

Negative symptoms and cognitive deficits are considered central components in schizophrenia, they are persistent over time, and have shown a poor response to pharmacological treatments, with executive deficits being those that have been most directly related to the prognosis and functioning of patients (Bagney et al., 2015).

Given the importance of the different regions of the PFC in the functioning of the EEFF, the study of the specific deficits in the *cool* and *hot* EEFF in patients with a predominance of negative schizophrenic symptoms, offers the possibility of investigating the relationship between the NS and the executive deficits, due to the *cool* and *hot* components have been associated with specific brain regions. Therefore if NSs and executive deficits share the same etiology, both being a clinical manifestation of dysfunction only in the dorsolateral region of the PFC, we would expect that patients with higher scores on the SANS scale will present a lower performance in the *cool* EEFF tasks. In the same way if the affectation of the NS was related to the ventromedial or orbitofrontal area, we would expect a higher score on the scale SANS will be associated with lower performance on *hot* EEFF tasks. However, in our study, the initial correlations found between the SANS scale score and the planning task did not survive after subsequent Bonferroni correction.

Similarly, authors such as Harvey et al. (2006) have proposed four theoretical models about the nature of the relationship between NSs and cognitive dysfunction, in the proposed models, these two dimensions could be either manifestations of the same basic process, or they could have characteristics independent, but with a similar underlying etiology. However, in our data we do not observe correlations that could indicate that both, NSs, and executive deficits are the product of the same basic process.

On the other hand, a third model postulates that both, NS and cognitive deficits will have a different etiology, but related to each other, this, due to factors such as the distributed neuropathology of the white matter, which would produce pathological changes in different brain regions causing the NS and the cognitive deficits. A last model would consider these two dimensions as different from each other and with different etiologies, attributing the correlations observed in the studies, to a problem of measurement and interpretation of the results. In this sense, the absence of correlations between NS and EEFF that we found in our data is a result congruent with these last two models, however, future studies of structural and functional changes of the brain are required, as well as longitudinal section correlation studies.

Regarding *dysexecutive syndrome*, in our study, we have applied the executive dysfunction subscale of the FrSBe scale, which assesses changes in behavior related to executive dysfunction or dysexecutive syndrome. Deficits on this scale have been linked to a malfunction in the dorsolateral prefrontal circuit (Grace and Malloy, 2001). However, we have not found correlations between their score and performance in the neuropsychological tasks, except in a first stay, between the executive dysfunction subscale and the latency times between movements in the short planning condition of the Tower of Hanoi. However, after Bonferroni’s subsequent correction, this finding has not survived.

A possible explanation for these results is that the deficits on this scale are deficits that are self-perceived by the patient, which may not be reflecting their actual functioning. In fact, various studies have reported a lack of awareness regarding the disease in these patients, being prevalent and more severe than in other mental pathologies (Garay Arostegui et al., 2014). Studies on disease awareness and cognitive decline in psychosis have concluded that these patients tend to obtain significantly lower scores in self-reflection, which could indicate this lack of awareness of the deficit, directly relating these difficulties to failures in the EEFF and especially with the deficiencies in working memory (Andreu Pascual et al., 2018).

## Limitations

Our findings must be interpreted in the context of various limitations.

First, although the study has a large battery of computerized neuropsychological tasks to evaluate the *cool* and *hot* executive functions, allowing us to have greater control regarding the presentation of stimuli and the collection of responses and thus minimizing the influence of evaluator biases, our study has not been carried out using the blind method, this because the recruitment and subsequent evaluation of the patients has been carried out in the hospital context, which in this case required the evaluator to know the clinical characteristics of the participant.

Second, we have a small number of participants, which could reduce the power of the study.

Third, although the scientific literature links an adequate functioning of the EEFF to the preserved prefrontal cortex, specifically the dorsolateral area for the *cool* executive functions and the ventromedial and orbitofrontal area with the *hot* EEFF, our study does not have physiological measures or brain neuroimaging measures that allow us to corroborate this hypothesis, so the use of these techniques would allow us to examine in a more direct way whether dysfunction in these neuroanatomical areas is related to an adequate functioning of executive functions.

Four, regarding the clinical variable of *pharmacological treatment*, the sample has not been divided according to the calculation of an estimate based on chlorpromazine equivalents.

## Implications and Future Research

Based on the analysis of the results obtained in this study, our findings showed that in psychotic patients with a predominance of NSs, both, the cognitive (*cool*) and emotional (*hot*) components of the EEFF are affected.

In reference to *cool* EEFF, some authors have suggested that the executive processes of WM (coding/maintenance and updating of information) and the ability to change the mental set, are primary (or central) executive components, than other more complex cognitive executive components as planning and troubleshooting are necessary for its proper functioning. In this sense, the deficiencies found in *cool* EEFF in the patients in our study could be related to the difficulties they have in planning and solving problems in their daily lives, leading to voluntary action disorders typical of these patients, thus leading to, to the poverty of action and perseverance they present.

In this sense, from the clinical point of view, the results found reinforce the need for a cognitive rehabilitation treatment of the executive components of WM and of the more complex cognitive components to obtain a clinical improvement in patients, which will allow them to perform your life in a more productive, adapted and satisfying way.

The difficulties found in *hot* EEFF, such as the recognition of emotions and ToM, could be at the base of the difficulties that these patients present in their abilities and social relationships; manifesting itself in an affectation of interpersonal relationships and diminished emotional behaviors that they present.

From the therapeutic point of view, these results guide us to work specifically with these patients in the recognition of emotions and rehabilitation in tasks typical of ToM. These aspects should be included in the psychotherapeutic approach to social skills training, an approach that in itself has shown evidence of its effectiveness. In fact, social skills training programs and different therapeutic approaches aimed at promoting social relationships contemplate, in one way or another, the aspects that are directed from emotional intelligence: emotional self-knowledge (perceive and understand emotions), emotional self-knowledge, regulation, personal motivation, empathy, and social relationships.

Finally, our results suggest with the compartmental data, that NSs in psychotic patients could be reflecting dysfunction both, in the dorsolateral region of the PFC and in the ventromedial and orbitofrontal regions.

Future research could examine the relationship between positive symptoms of schizophrenia and performance in a similar battery of neuropsychological tasks, which assesses both, the cognitive area and its most socio-emotional part, this approach can help us to understand the variety of deficits observed in the schizophrenia providing specific patterns of association between disease symptoms and neuropsychological profiles.

On the other hand, although we understand the limitations of not using neuroimaging techniques in this study, we believe that a similar behavioral approach in further investigations that study the positive symptoms of schizophrenia can also provide interesting data to contrast with those found in this study.

Similarly, in future research, and due to the importance of medication in the cognition of patients, the effects of medication should be analyzed from methods that allow the standardization of doses of antipsychotics, such as chlorpromazine equivalents, or the Defined daily dose system (DDD) (Nosè et al., 2008).

Having more knowledge at this level will help to adjust the psychotherapeutic and cognitive treatments and/or intervention programs aimed at these patients, while improving our knowledge about the behavioral, cognitive, and emotional manifestations of the disease.

## Data Availability Statement

The raw data supporting the conclusions of this article will be made available by the authors, without undue reservation.

## Ethics Statement

The studies involving human participants were reviewed and approved by Healthcare Ethics Committee (CEA), Almería Centro. The patients/participants provided their written informed consent to participate in this study.

## Author Contributions

PR-C, MD, and ES-M conceived and designed the experiments, interpreted the data, and wrote the first draft of the manuscript. HA-L performed the statistical analysis. PR-C, MD, ES-M, and HA-L approved the final manuscript. All the authors contributed to the article and approved the submitted version.

## Conflict of Interest

The authors declare that the research was conducted in the absence of any commercial or financial relationships that could be construed as a potential conflict of interest.
